# Modulatory Effect of Nicotinic Acid on the Metabolism of Caco-2 Cells Exposed to IL-1β and LPS

**DOI:** 10.3390/metabo10050204

**Published:** 2020-05-16

**Authors:** Maria Laura Santoru, Cristina Piras, Federica Murgia, Martina Spada, Laura Tronci, Vera Piera Leoni, Gabriele Serreli, Monica Deiana, Luigi Atzori

**Affiliations:** Department of Biomedical Sciences, University of Cagliari, Metropolitan City of Cagliari, 09042 Monserrato, Italy; cristina.piras@unica.it (C.P.); federica.murgia@unica.it (F.M.); martina.spada@unica.it (M.S.); lauratronci90@gmail.com (L.T.); vera.leoni@tiscali.it (V.P.L.); gabrieleserreli@hotmail.it (G.S.); mdeiana@unica.it (M.D.); latzori@unica.it (L.A.)

**Keywords:** inflammation, metabolomics, IBD, nicotinic acid

## Abstract

Inflammatory bowel diseases (IBD) are the most common gastrointestinal inflammatory pathologies. Previous work evidenced a lower content of nicotinic acid (NA) in feces of IBD patients compared to healthy subjects. In the present study, we aimed to understand the effects of NA on intestinal inflammation, as several studies reported its possible beneficial effect, and investigate its influence on inflammation-driven metabolism. NA was tested on a Caco-2 in-vitro model in which inflammation was induced with interleukin-1β (IL-1β) and lipopolysaccharide (LPS), two mayor proinflammatory compounds produced in IBD, that stimulate the production of cytokines, such as interleukin 8. A metabolomics approach, with gas chromatography–mass spectrometry (GC-MS) and nuclear proton magnetic resonance (^1^H-NMR), was applied to study the metabolic changes. The results showed that NA significantly reduced the level of IL-8 produced in both LPS and IL-1β stimulated cells, confirming the anti-inflammatory effect of NA also on intestinal inflammation. Moreover, it was demonstrated that NA treatment had a restoring effect on several metabolites whose levels were modified by treatments with IL-1β or LPS. This study points out a possible use of NA as anti-inflammatory compound and might be considered as a promising starting point in understanding the beneficial effect of NA in IBD.

## 1. Introduction

Inflammatory bowel disease (IBD) is a chronic inflammatory disease responsible for profound changes in the gastrointestinal tract. These changes are due, at least in part, to the recruitment of inflammatory cells, particularly myeloid cells such as neutrophils and monocytes, and include exhaustion of nutrients, increased oxygen consumption, and generation of large quantities of reactive nitrogen and oxygen intermediate [1]. The dysregulation and the inappropriate response of the immune system against the microflora of the gut are typical features of the IBD, too. Another important feature of gut inflammation is the disruption of the homeostasis between microbes and host, the so-called dysbiosis [2]. Upon activation of the immune system, cytokines and chemokines, are produced [3], triggering a cascade of downstream reactions [4]. Most of the changes happening during these processes are well known, but some still need to be fully elucidated, especially those responsible for the metabolic alteration. Different cytokines produced during intestinal inflammation seem to have a direct effect on metabolism. One of the most important cytokines secreted is interleukin-1β (IL-1β). This cytokine is responsible for the activation of lipolysis, inhibition of gluconeogenesis, and increase of vascular permeability to fluids and solutes [3]. Considering the preponderance of Gram negative bacteria in the gastrointestinal tract, lipopolysaccharide (LPS) plays a crucial role in the activation of immune response and consequently in the progression of the disease [5]. Moreover, LPS can stimulate the release of interleukin 8 (IL-8) and other inflammatory cytokines in different cell types, leading to an acute inflammatory response [6]. The influence on metabolism carried on by the inflammatory process is particularly important when talking about the gastrointestinal tract [7]. Indeed, the intestinal epithelium is composed of a very dynamic barrier that is regulated in a complex way to both accommodate the transport of nutrients and fluids, and then select and exclude any antigens from the luminal interface [8]. In our previous work, we applied a multi-omics approach, both metabolomic and metagenomic, to study feces samples coming from IBD patients [9]. This study evidenced a low content of NA in feces of patients affected by IBD, suggesting an anti-inflammatory role of NA. Indeed, as NA seems to exert anti-inflammatory effects in different tissues [10,11], in the present study we aimed to investigate its effect on intestinal inflammation and metabolism using an inflammation in-vitro model based on Caco-2 cells stimulated with IL1-β and LPS [12].

## 2. Results

### 2.1. Evaluation of Cell Viability

**Figure 1:**
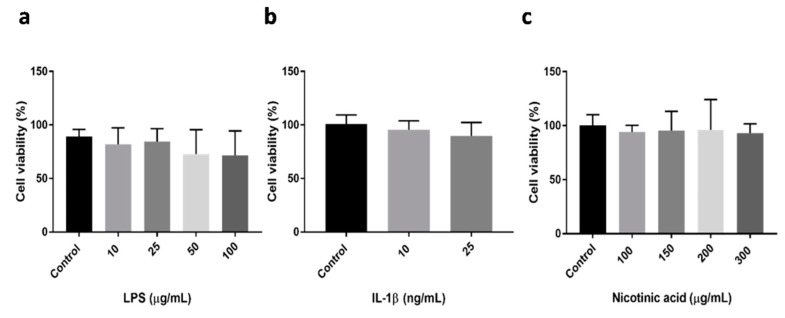
Effects of LPS (**a**), IL-1β (**b**), and NA (**c**) on cell viability. Cell viability was evaluated after 48 h of incubation of each compound. Data, expressed as % of control, are presented as means ± standard deviation. Statistical analysis was performed using an unpaired Student’s *t*-test.

To investigate the effect of LPS, IL-1β, and NA on differentiated Caco-2 cell monolayers, experiments were initially carried out to assess cell viability after treatment and to choose a non-toxic concentration for each compound. As reported in Figure 1, cell viability remained unchanged in the presence of the tested compounds. LPS was tested in the concentration range 10–100 μg/mL, IL-1β in a concentration of 10 and 25 ng/mL and NA in the concentration range 100–300 μg/mL. The cell viability was tested after 48 h of treatment for each compound.

### 2.2. Evaluation of IL-8 Production

**Figure 2:**
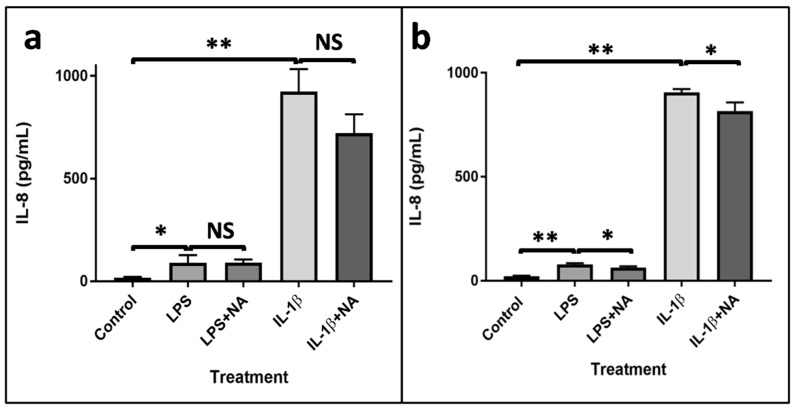
IL-8 measurement. Detection of IL-8 levels in Caco-2 cell culture medium after treatment with LPS (50 µg/mL) and LPS (50 µg/mL) +NA (100 µg/mL) and after treatment with IL-1β (25 ng/mL) and IL-1β (25 ng/mL)+NA (100 µg/mL) (**a**) and detection of IL-8 levels in Caco-2 cell culture medium after treatment with LPS (50 µg/mL) and LPS (50 µg/mL) +NA (200 µg/mL) and with IL-1β (25 ng/mL) and IL-1β (25 ng/mL) +NA (200 µg/mL) (**b**). Statistical analysis was performed by a Student’s *t*-test. Results were considered significant when * *p* < 0.05 and ** *p* < 0.01. NS = Non-significant.

To estimate the effect of LPS, IL-1β, and NA on inflammation, an ELISA assay was carried out to measure the levels of IL-8 released after treatments with each compound. In the first experiment, cells were treated with LPS (50 µg/mL) and LPS in combination with NA (100 µg/mL) and with IL-1β (25 ng/mL) and IL-1β plus NA (100 µg/mL; Figure 2a). As shown in the figure, the levels of IL-8 were significantly increased after treatment with both LPS and IL-1β, while NA at the concentration of 100 µg/mL did not reduce the production of IL-8 when compared with LPS and IL-1β alone. Therefore, a second experiment was performed with a higher concentration of NA (200 µg/mL). As shown in Figure 2b, LPS and IL-1β increased IL-8 production, and NA reduced the IL-8 levels. 

### 2.3. Metabolomics Analysis 

**Figure 3:**
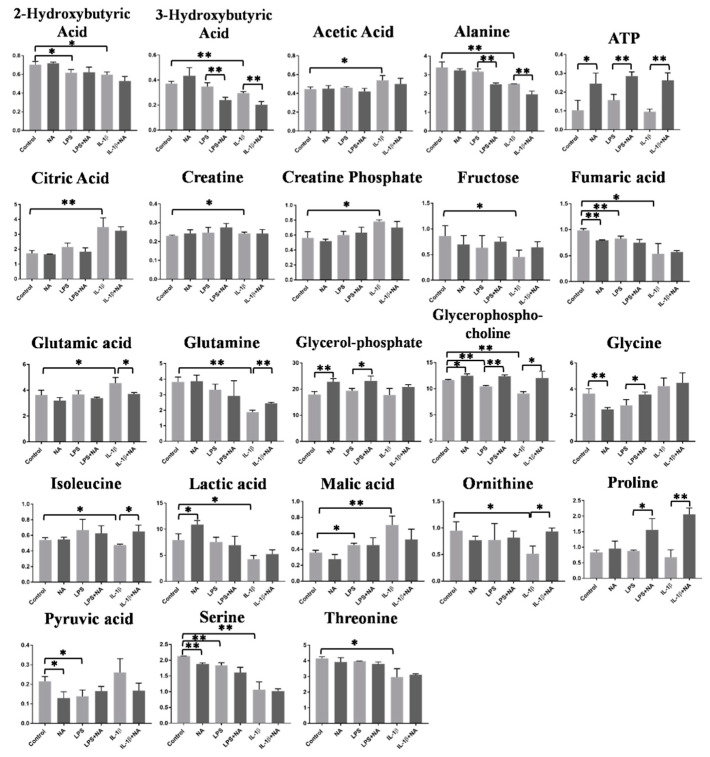
Significantly different intracellular metabolites measured in Caco-2 cells by ^1^H-NMR and GC-MS. Metabolites are indicated as peak areas normalized for total protein content and total area (*n* = 3). Statistical analysis was performed using an unpaired Student’s *t*-test. * *p* < 0.5, ** *p* < 0.01.

To investigate the metabolic changes induced by NA, Caco-2 cells were exposed to LPS (50 µg/mL), IL-1β (25 ng/mL), and NA (200 µg/mL) alone or in combination for 48 h. Cells and cell culture media metabolites extracts were analyzed with both GC-MS and ^1^H-NMR, two of the most common techniques used in metabolomics. A total of 47 compounds were identified and quantified with the NMR approach in cells and 21 compounds in media, while 44 and 34 compounds were identified respectively in cells and media with the GC-MS (supporting Figures S1–S8 and supporting Table S1). Metabolomics results show that IL-1β had a remarkable effect on energetic metabolism, compared to the LPS effect on the same metabolic pathways (Figure 3, Table 1): intracellular levels of acetic acid, glutamic acid, citric acid, creatine, creatine phosphate, and malic acid were significantly increased after the 48 h treatment with IL-1β, while 2-hydroxybutyric acid, 3-hydroxybutyric acid, alanine, fructose, fumaric acid, glutamine, threonine, isoleucine, lactic acid, ornithine, serine, and glycerophosphocholine were decreased. On the other hand, the LPS treatment induced an increase in malic acid levels and a decrease in 2-hydroxybutyric acid, fumaric acid, serine, glycerophosphocholine, and pyruvic acid. Extracellular metabolites were measured in the cell culture medium.

**Figure 4:**
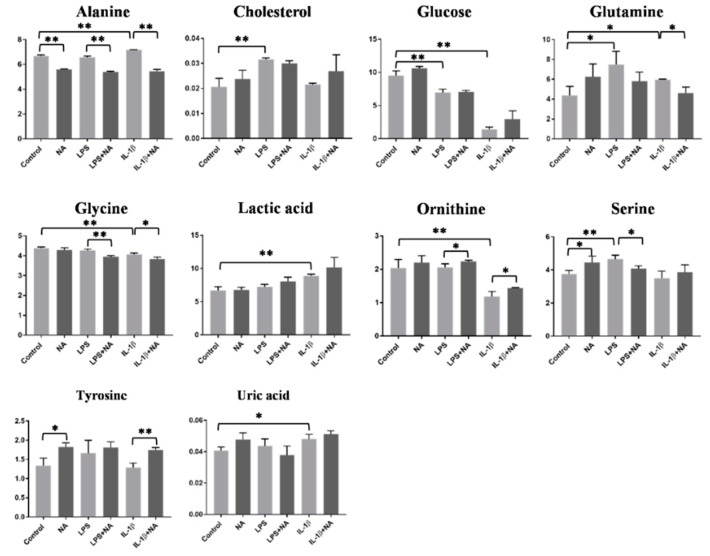
Significantly different extracellular metabolites measured in the medium of Caco-2 cell by ^1^H-NMR and GC-MS. Metabolites are presented as peak areas normalized for total protein content and total area (*n* = 3). Statistical analysis was performed using an unpaired Student’s *t*-test. * *p* < 0.05, ** *p* < 0.01.

Results show that alanine, glutamine, lactic, and uric acid were increased in the cell culture medium after IL-1β treatment, showing an opposite trend compared to the intracellular levels. Conversely, glycine, ornithine, and glucose levels were decreased. As IL-1β, LPS increased serine, glutamine, and cholesterol levels in the cell culture medium while glucose levels were decreased. (Figure 4, Table 1). 

The effect of NA treatment alone on Caco-2 model was also investigated. NA treatment alone caused the increase of ATP, glycerolphosphate, lactic acid, and glycerophosphocholine and a decrease of fumaric acid, glycine, pyruvic acid, and serine at the intracellular level (Figure 3, Table 1), while at the extracellular level, there were increased levels of serine and tyrosine and lower levels of alanine (Figure 4, Table 1).

Regarding the effects of NA on rescuing metabolism after inflammation, levels of glutamine, isoleucine, ornithine, and glycerophosphocholine were increased and glutamic acid levels were reduced after IL-1β+NA treatment compared to the IL-1β treatment alone (Figure 3, Table 1). 

**Figure 5:**
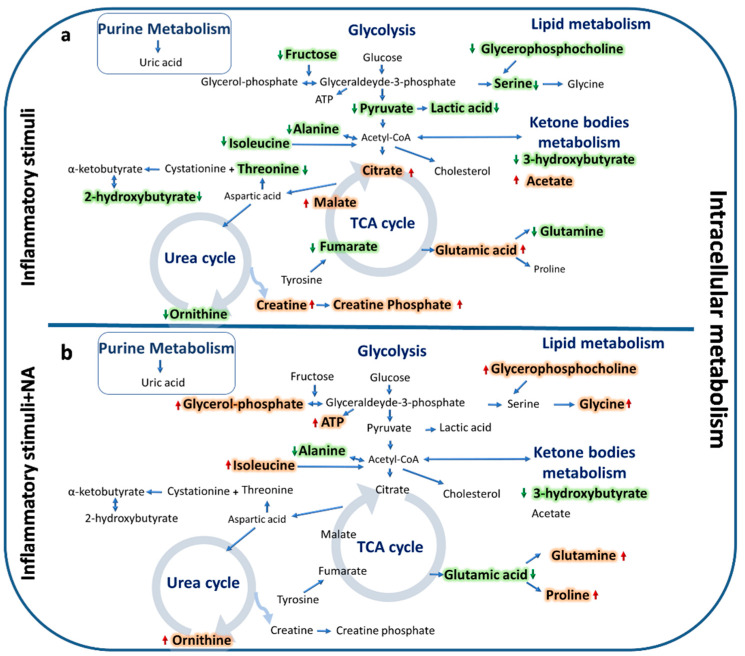
Relevant metabolic pathways that were found significantly altered after treatment with proinflammatory stimuli (LPS and IL-1β) (**a**) and after treatment with proinflammatory stimuli in combination with NA (**b**) in Caco-2 cells at the intracellular level. Increased and decreased metabolites are highlighted in red and green, respectively.

Moreover, at the intracellular level, the IL-1β+NA treatment was able to reduce levels of alanine, 3-hydroxybutyric acid and to increase ATP and proline levels when compared to the IL-1β treatment alone. The treatment with LPS+NA caused an increase in the levels of glycerophosphocholine, ATP, glycerolphosphate, glycine, and proline and a decrease of 3-hydroxybutyric acid and alanine levels, compared to LPS treatment alone at the intracellular level (Figure 3, Table 1). At the extracellular level, results showed a decrease of alanine and glutamine, contrarily to ornithine and tyrosine, which were increased in IL-1β+NA treated samples compared to IL-1β treatment alone. At the extracellular level, LPS+NA treatment also induced a decrease of serine, alanine, and glycine and an increased level of ornithine when compared to LPS alone (Figure 4, Table 1). Overall, metabolites found statistically perturbed in stimulated Caco-2 cells were mostly associated with energetic pathways (such as glycolysis, tricarboxylic acid (TCA) cycle, urea cycle, ketone bodies metabolism, and lipid metabolism) at the intracellular level (Figure 5a). After IL-1β and LPS stimuli plus NA the same pathways appeared to be modified, but in the opposite direction (Figure 5b). 

## 3. Discussion

The metabolic profile is altered in conditions of active inflammation, such as those associated with IBD and has become an area of significant interest for research [8]. Our previous work pointed out several metabolites altered by the inflammation process in patients with IBD [9]. Among all altered metabolites, NA levels were found to be lower in the feces of these patients. NA, also known as niacin or vitamin B3, is a water-soluble vitamin whose derivatives, such as NADH, NAD^+^, NADPH, and NADP^+^, play essential roles in energy metabolism in the living cell [13]. The designation vitamin B3 also includes the amide form, nicotinamide, or niacinamide. Severe lack of niacin causes deficiency disease pellagra, whereas a mild deficiency slows down the metabolism decreasing cold tolerance [14]. Among all the effects of NA, different studies proved its role in ameliorating the inflammatory process [15,16]. Moreover, it has been demonstrated that nicotinic acid metabolites, such as nicotinamide, have an antioxidant and anti-inflammatory effect, on human primary monocytes and monocyte-derived macrophages [17] and it has been evidenced that these metabolites could interfere with early events associated to the differentiation of monocytic cell line THP-1 and that they affect LPS-induced biological responses of the cell line [18]. In the present study, an in vitro model was used to study NA effects on intestinal inflammation. Differentiated Caco-2 cells were used and inflammation was induced with the two major players in intestinal inflammation, LPS and IL-1β. LPS is a toxic component of the outer membrane of Gram negative bacteria and it is a potent initiator of inflammation [5,6,19]. Indeed, LPS induces systemic inflammatory injury and various pathological changes [20]. As known, LPS can stimulate Caco-2 cells proliferation [21], but in our study we used a wide and common model of differentiated Caco-2 cells, in which cells are seeded and kept in culture for 15 days before each treatment. During these days, the cells differentiate, reach the confluency and form a monolayer, so that their possible further growth is arrested. Among cytokines, IL-1β seems to play a particularly important role in intestinal inflammation as several clinical studies have reported high levels of IL-1β secretion by colon lamina propria monocytes from patients with active IBD [22,23]. In the present study, we used LPS and IL-1β as inflammation stimuli, assessing effective, but not lethal, concentration by performing an MTT assay. A high dose of LPS (50 µg/mL) was used to induce IL-8 production and to avoid statistically significant cell death. Moreover, a concentration of 50 µg/mL or higher of LPS was used to induce an inflammatory response in Caco-2 cells in several studies [24,25]. Stimulation of inflammation was evaluated by measuring IL-8 production after treatment with LPS and IL-1β at 50 µg/mL and 25 ng/mL respectively. Indeed, one of the earliest reported chemokines, produced by intestinal epithelial cells, is the IL-8 [26]. Similar to our findings, IL-8 mRNA levels were significantly higher in inflamed mucosa of IBD patients than in not inflamed mucosa [27]. Furthermore, our findings reported that the production of IL-8 is higher after IL-1β treatment compared to LPS. Our results revealed an altered metabolism as an effect of inflammation. Both LPS and IL-1β had a similar effect on a pattern of metabolites resulting in the alteration of energetic pathways, such as TCA cycle, glycolysis, urea cycle, and lipid metabolism. It has already been reported that ongoing inflammatory and immune responses are associated with dramatic shifts in tissue metabolism [1]. Particularly, a significant metabolic change during inflammation and within the immune response involves the generation of lipid mediators and glycolysis. Indeed, impaired glycolysis is a hallmark of inflammatory cells [28] as shown also by our results. This confirms the state of inflammation induced in these cells after been treated with both IL-1β and LPS, suggesting that this model may be reliable to study inflammatory mechanisms. The results indicate that NA can reduce inflammation in the enterocytes and this effect is dose-dependent. Our data are in agreement with previous studies showing anti-inflammatory effects of NA in in vitro and in vivo models [16,29]. In the present study, we also evaluated the effect of NA on normal and inflammation related metabolism. NA seems to influence Caco-2 cell metabolism. Indeed, comparing NA non-treated and NA treated cells there was an increase of ATP, lactic acid, glycerol-phosphate, and glycerophosphocholine and a decrease of fumaric acid and pyruvic acid at the intracellular level and an increase of serine and tyrosine and a decrease of alanine at the extracellular side. The increase of ATP levels may indicate a higher energetic metabolism. These data may help to understand and clarify the effects of NA on lipids and glucose metabolism described in the literature [30]. As mentioned before, the effects of NA on the inflammation metabolism were evaluated as well. Interestingly, from the results obtained in the present study, NA treatment seemed to have a restoring effect on different metabolites. For example, at the intracellular side, the level of glutamic acid, which was upregulated by IL-1β treatment, was then decreased comparing it with IL-1β plus NA treated cells. On the other hand, levels of glutamine, isoleucine, ornithine, and glycerophosphocholine, which were downregulated by IL-1β, were increased in the comparison between IL-1β and IL-1β plus NA treated cells. A similar trend was confirmed at the extracellular level, where the levels of alanine and glutamine that were increased by IL-1β were then decreased by NA treatment, and the ornithine level that was downregulated by IL-1β was increased by NA. Looking at the effect of NA on LPS induced inflammation, it is possible to observe again the restoring effect of NA, as it was able to increase the level of glycerophosphocholine, which was decreased by LPS treatment at the intracellular side, and to decrease the level of serine, which was increased by LPS alone.

## 4. Materials and Methods

### 4.1. Cell Culture

Caco-2 cells (ECACC Salisbury, Wiltshire UK) were cultured in Dulbecco’s modified Eagle’s medium low glucose with L-glutamine and sodium pyruvate (Euroclone, Milan, Italy) supplemented with 10% heat-inactivated bovine serum, 100 U/mL penicillin, and 100 mg/mL streptomycin. Cells were cultured in monolayers, at 37 °C, in a humidified atmosphere of 5% CO_2_, replacing the medium twice a week. 

### 4.2. MTT Viability Test

The MTT assay was assessed on Caco-2 cells to evaluate the viability of the cells in the presence of the tested compounds. Cells were seeded in 96-well plates (5 × 10^4^ cells/mL in 100 μL) and exposed to different concentrations of the compounds alone: 10, 25, 50 and 100 µg/mL for LPS from *Escherichia coli* O111:B4 (Sigma-Aldrich, Milan, Italy), 10 and 25 ng/mL for IL-1β (Merck, Rome, Italy) and 100, 150, 200 and 300 µg/mL for NA (Sigma-Aldrich, Milan, Italy). NA was dissolved in the cell medium. After 48 h incubation, the medium was removed and 100 μL of MTT solution (2.5 mg/mL in fresh medium) were added and left for 2 h at 37 °C. Formazan crystals were then solubilized by adding 100 μL of dimethyl sulfoxide (DMSO). The absorbance was read at 570 nm using a microplate reader (Infinite 200, Tecan, Salzburg, Austria).

### 4.3. Treatments

For experimental studies, Caco-2 cells were plated in Petri dishes and used 15–17 days post-seeding, when fully differentiated [31]. To induce the inflammation process, cells were stimulated with two different compounds: LPS and IL-1β. The effects of NA were then studied by pretreating cells for 24 h with NA, and then stimulated with both NA plus LPS and NA. At the end of the experiment, cells and media were collected for further analysis.

### 4.4. Evaluation of IL-8 Protein Levels

An aliquot of 150 µL of cell culture medium was collected from the Petri dishes and used for ELISA detection. Levels of IL-8 were quantified using the Human IL-8 ELISA kit (CliniScinces, Nanterre, France) and following the manufacturer’s instructions. Sample absorbance values were read at 450 nm in a microplate reader (Infinite 200, Tecan, Salzburg, Austria).

### 4.5. Cell Culture Samples Preparation for Metabolomics Analysis

To perform metabolomics analysis, the growth medium was removed from the Petri dishes and aliquoted (500 μL) in eppendorf tubes to be treated similarly to the cells. Cells were washed with 3 mL of physiological solution, and intracellular metabolites were extracted with 1.2 mL of cold methanol/water (80:20) and shacked for 15 min at low temperature (4 °C). Then cells were harvested by scraping and transferred in eppendorf tubes. To ensure the complete lysis of the cells, the extraction was combined with 10 min of ultrasonic treatment at a controlled temperature (4 °C). The growth medium was extracted as described above. Briefly, 500 μL of medium were aliquoted in eppendorf tubes and centrifuged at 5500× g, 10 min, 4 °C, to remove cell debris and dead cells. Then 1.2 mL of cold methanol/water with the internal standard (80:20) was added to the supernatant of the medium and the extraction procedure followed as described for the cells. Cell suspensions and growth medium were centrifuged at 5500× *g* for 30 min at 4 °C. For GC-MS analysis, 400 μL of supernatant was aliquoted and dried in an Eppendorf^TM^ Concentrator Plus overnight. Dried pellets were derivatized with 50 μL of a solution of methoxamine in pyridine (10 mg/mL; Sigma-Aldrich, St. Louis, MO, USA). After 1 h at 70 °C, 50 μL of MSTFA (Sigma-Aldrich, St. Louis, MO, USA) were added and left at room temperature for one hour. Successively, 50 μL of hexane were added and samples were transferred in a vial for the GC-MS analysis. A pool of all samples was created and used as quality control (QC.) For ^1^H-NMR analysis, 700 μL of supernatant was aliquoted and dried in an Eppendorf^TM^ Concentrator Plus overnight. Dried hydrophilic cells and medium extracts were redissolved in 690 μL of potassium phosphate buffer in D2O (100 mM, pH 7.4) and 10 μL of TSP (sodium 3-trimethylsilyl-propionate-2,2,3,3,-d4) as a chemical shift reference (δ 0.0; 98 atom % D, Sigma-Aldrich, St. Louis, MO, USA). An aliquot of 650 μL was analyzed by ^1^H-NMR. 

### 4.6. Protein Extraction and Quantification

Total proteins were extracted with the Bradford assay, with some modifications. Cell pellets were added with 500 μL Cell Lytic M lysis buffer (Sigma-Aldrich, Milan, Italy) for protein extraction, supplemented with mammalian protease and phosphatase inhibitor cocktail (1:100 *v*/*v*). Cells were scraped on ice, incubated for 15 min on ice, centrifuged at 12,500× *g* at 4 °C for 7 min and then stored at −20 °C prior to quantification. The calibration curve was generated using bovine serum albumin (BSA) in Cell Lytic M reagent, at different standard concentrations (0.1–5 mg/mL). 500 μL of Bradford Reagent solution (Sigma-Aldrich, St. Louis, MO, USA) were added and incubated with 10 μL of standard and protein sample at room temperature for 5 min. The absorbance was measured at 595 nm using a microplate reader (Infinite 200, Tecan, Salzburg, Austria) at a controlled temperature of 37 °C. 

### 4.7. Gas Chromatography–Mass Spectrometry Analysis

Derivatized samples were injected splitless into a 7890A gas chromatograph coupled with a 5975C network mass spectrometer (Agilent Technologies, Santa Clara, CA, USA) equipped with a 30 m × 0.25 mm ID, fused silica capillary column, with a 0.25 μM TG-5MS stationary phase (Thermo Fisher Scientific, Waltham, MA, USA). The injector and transfer line temperatures were at 250 °C and 280 °C, respectively. The gas flow rate through the column was 1 mL/min. The column initial temperature was kept at 60 °C for 3 min, then increased to 140 °C at 7 °C/min, held at 140 °C for 4 min, increased to 300 °C at 5 °C/min, and kept for 1 min. Identification of metabolites was performed using the standard NIST 08 and GMD mass spectra libraries and, when available, by comparison with authentic standards. Peak detection and deconvolution, filtering, and normalization were performed using a pipeline on Knime [32]. The total protein content together with the total area of chromatograms were used to normalize the metabolites measurements of each cell and medium sample.

### 4.8. ^1^H-NMR Measurements

^1^H-NMR analysis was performed using a Varian UNITY INOVA 500 spectrometer operating at 499.839 MHz for proton and equipped with a 5 mm double resonance probe (Agilent Technologies, CA, USA). ^1^H-NMR spectra were acquired at 300 K with a spectral width of 6000 Hz, a 90° pulse. The acquisition time was of 1.5 s, the relaxation delay was of 2 s, and for each sample, 512 FID were collected into 64 K data points. The residual water signal was suppressed by applying a presaturation technique with low power radiofrequency irradiation for 2 s. After Fourier transformation with 0.3 Hz line broadening and a zero-filling to 64 K, ^1^H-NMR spectra were manually phased and baseline corrected using ACD Lab Processor Academic Edition (Advanced Chemistry Development, 12.01, 2010). Spectral chemical shift referencing on the TSP CH3 signal at 0.00 ppm was performed on all spectra. Metabolites were identified and quantify of each NMR spectra of samples using the Chenomx NMR Suite 7.1 (Chenomx Inc., Edmonton, Alberta, Canada) [33]. The total protein content together with the total area of spectra was used to normalize the metabolites measurements of each cell and medium sample.

### 4.9. Univariate Statistical Analysis

GraphPad Prism software (version 7.01, GraphPad Software, Inc., CA, USA) was used to perform the univariate statistical analysis on the data. Specifically, a Student’s *t*-test was performed to test statistical significance. All experimental data are the mean of three experiments.

## 5. Conclusions

In conclusion, this in vitro study demonstrated that NA could exert a counteracting effect in intestinal inflammation, by reducing IL-8 production after inflammatory stimuli. Furthermore, data presented here also showed how NA plays a role in metabolic rewiring, by restoring metabolites levels, altered in the inflammation process. Taken together, our data pinpointed a potential strategy to counteract intestinal inflammation, although, future studies are needed to clarify the mechanism behind the NA effects in IBD. Surely, to test the real potentiality of NA as an anti-inflammatory compound in IBD it should be tested as well in in-vivo and human studies.

## Figures and Tables

**Table 1:** Intracellular and extracellular metabolites significantly altered by treatment with proinflammatory stimuli (LPS and IL-1β) and by treatment with proinflammatory stimuli in combination with NA in Caco-2 cells.

**Intracellular Metabolites**			
**Proinflammatory Stimuli**		**Proinflammatory Stimuli + NA**	
2-Hydroxybutyrate	↓	-	
3-Hydroxybutyrate	↓	3-Hydroxybutyrate	↓
Acetate	↑	-	
Alanine	↓	Alanine	↓
-		ATP	↑
Citrate	↑	-	
Creatine	↑	-	
Creatine Phosphate	↑	-	
Fructose	↓	-	
Fumarate	↓	-	
Glutamic acid	↑	Glutamic acid	↓
Glutamine	↓	Glutamine	↑
-		Glycerolphosphate	↑
Glycerophosphocholine	↓	Glycerophosphocholine	↑
-		Glycine	↑
Isoleucine	↓	Isoleucine	↑
Lactic acid	↓	-	
Malate	↑	-	
Ornithine	↓	Ornithine	↑
-		Proline	↑
Pyruvate	↓	-	
Serine	↓	-	
Threonine	↓	-	
			
**Extracellular Metabolites**			
**Proinflammatory Stimuli**		**Proinflammatory Stimuli + NA**	
Alanine	↑	Alanine	↓
Cholesterol	↑	-	
Glycine	↓	Glycine	↓
Glucose	↓	-	
Glutamine	↑	Glutamine	↓
Lactic acid	↑	-	
Ornithine	↓	Ornithine	↑
Serine	↑	Serine	↓
-		Tyrosine	↑
Uric acid	↑	-	

## Supplementary Materials

The following are available online at https://www.mdpi.com/2218-1989/10/5/204/s1.
