# Supplementary material for: Modulatory Effect of Nicotinic Acid on the Metabolism of Caco-2 Cells Exposed to IL-1β and LPS
Source: Metabolites. 2020 May 16;10(5):204. doi: 10.3390/metabo10050204 (PMC7281454; doi:10.3390/metabo10050204)
Supplement: Supplementary file 1 [file metabolites-10-00204-s001.pdf]

^{1}H-NMR spectra

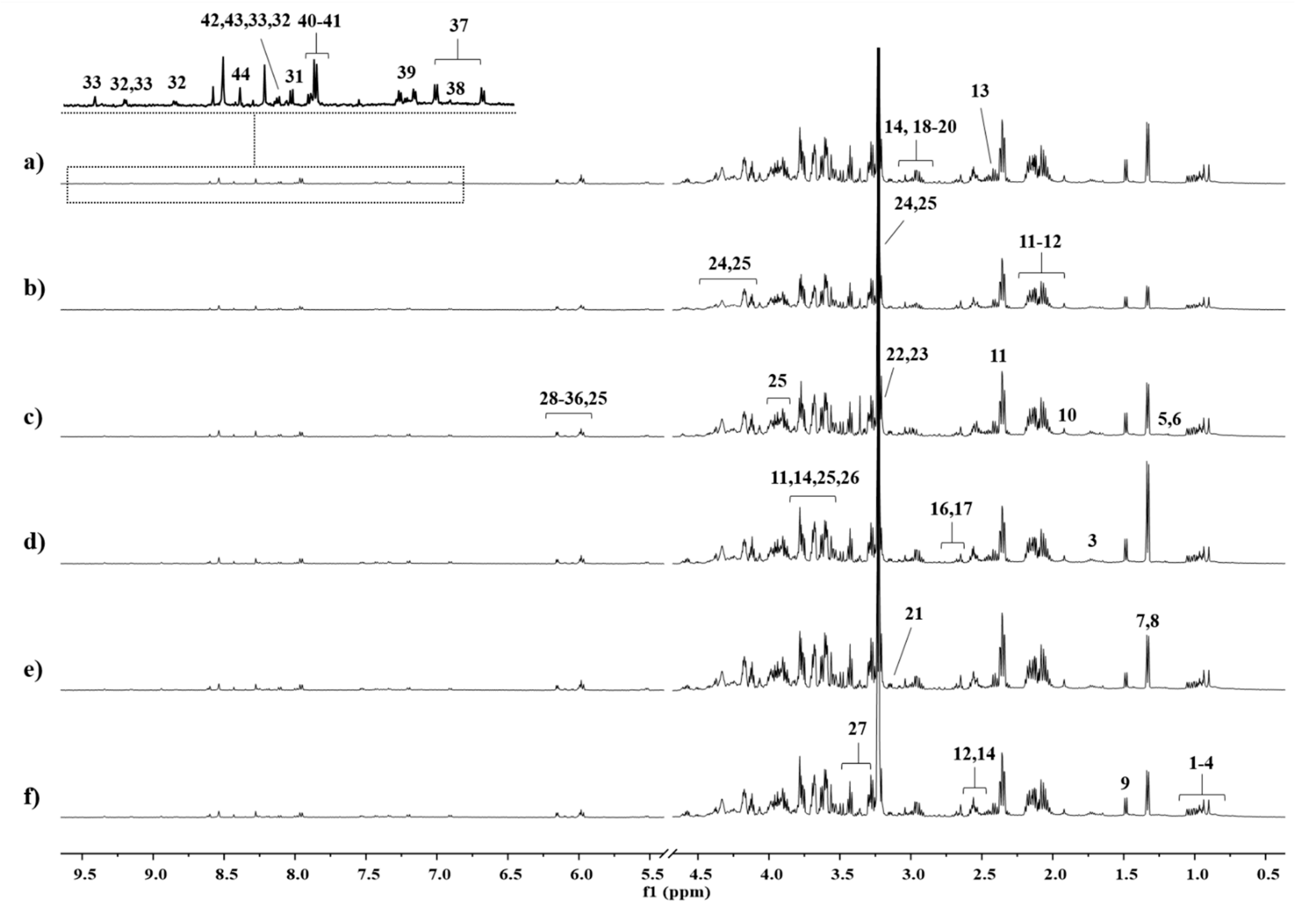

Figure S1. 500 MHz 1H NMR spectra of Caco-2 cells extracts obtained from a) control, b) after treatment with IL-1β, c) after treatment with LPS, d) after treatment with NA, e) after treatment with IL-1β + NA, f) after treatment with LPS + NA. Peaks: 1) Pantothenate 2) Isoleucine 3) Leucine 4) Valine 5) 3-Hydroxyisobutyrate 6) Ethanol 7) Lactate 8) 2-Hydroxyisobutyrate 9) Alanine 10) Acetate 11) Glutamate 12) Glutamine 13) 3-Hydroxy-3-methylglutarate 14) Glutathione 15) Isocitrate 16) Methionine 17) Aspartate 18) Creatine 19) Creatine phosphate 20) Creatinine 21) Ethanolamine 22) Choline 23) O-acetylcarnitine 24) O-phosphocholine 25) Glycero-3-phosphocholine 26) Glucosio-1-phosphate 27) Taurine 28) UDP-acetylglucosamine 29) UDP-glucose 30) UDP-glucuronate 31) UMP 32) Nicotinic acid 33) NAD+/NADH/ NADP+ 34) ADP 35) GTP 36) ATP 37) Tyrosine 38) Histidine 39) Phenylalanine 40) Riboflavin 41) Xanthine 42) Guanosine 43) Anserine 44) Imidazole 45) Formate.

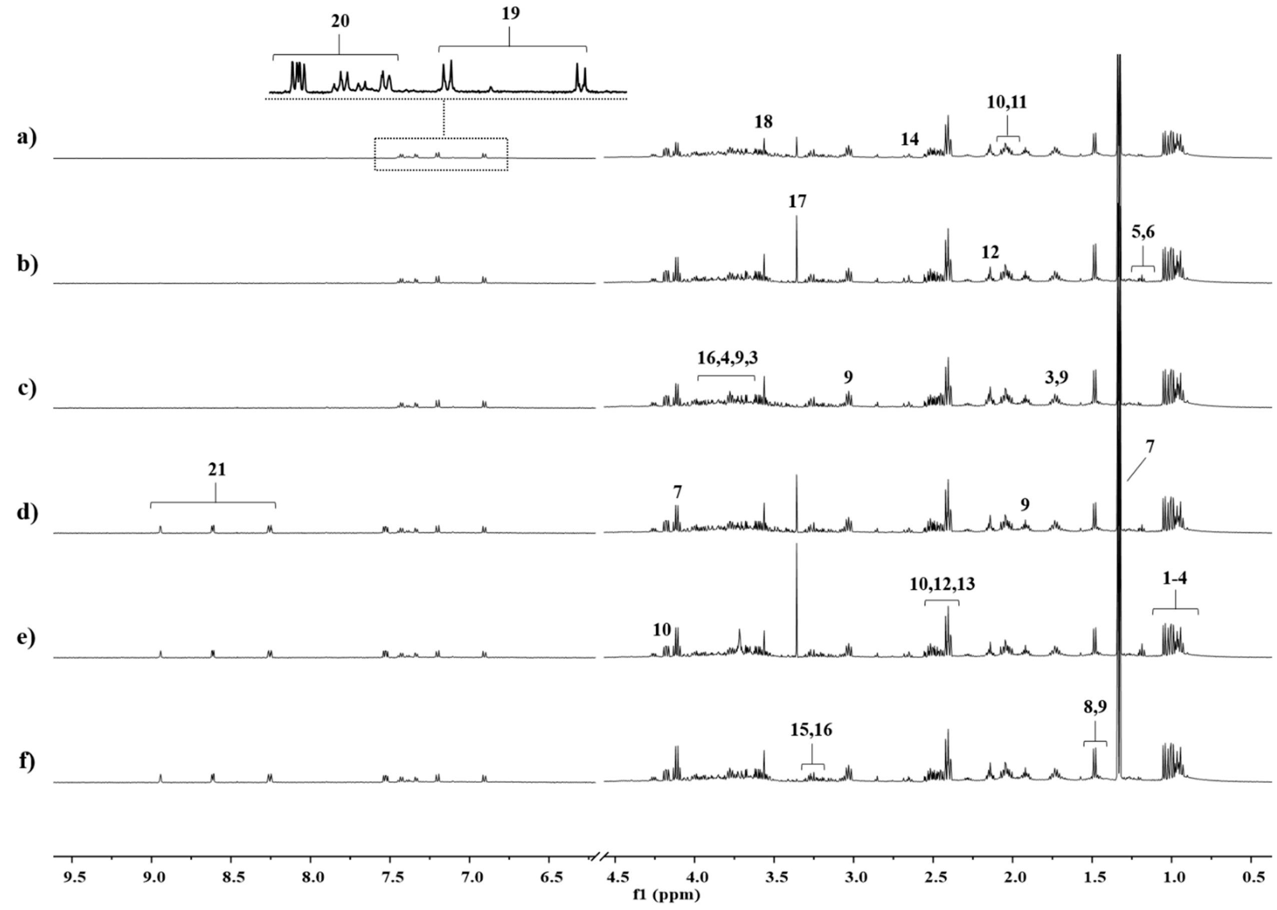

Figure S2. **Figure S2**. 500 MHz ^{1}H NMR spectra of Caco-2 cells medium extracts obtained from **a)** control, **b)** after treatment with IL-1β, **c)** after treatment with LPS, **d)** after treatment with NA, **e)** after treatment with IL-1β + NA, **f)** after treatment with LPS + NA. **Peaks**: 1) Pantothenate 2) Isoleucine 3) Leucine 4) Valine 5) 3-Hydroxyisobutyrate 6) Ethanol 7) Lactate 8) Alanine 9) Lysine 10) Pyroglutamate 11) N-acetylglucosamine 12) Glutamine 13) N-acetylglutamine 14) Methionine 15) cis-Aconitate 16) Myo-inositol 17) Methanol 18) Glycine 19) Tyrosine 20) Phenylalanine 21) Nicotinate.

GC-MS chromatograms

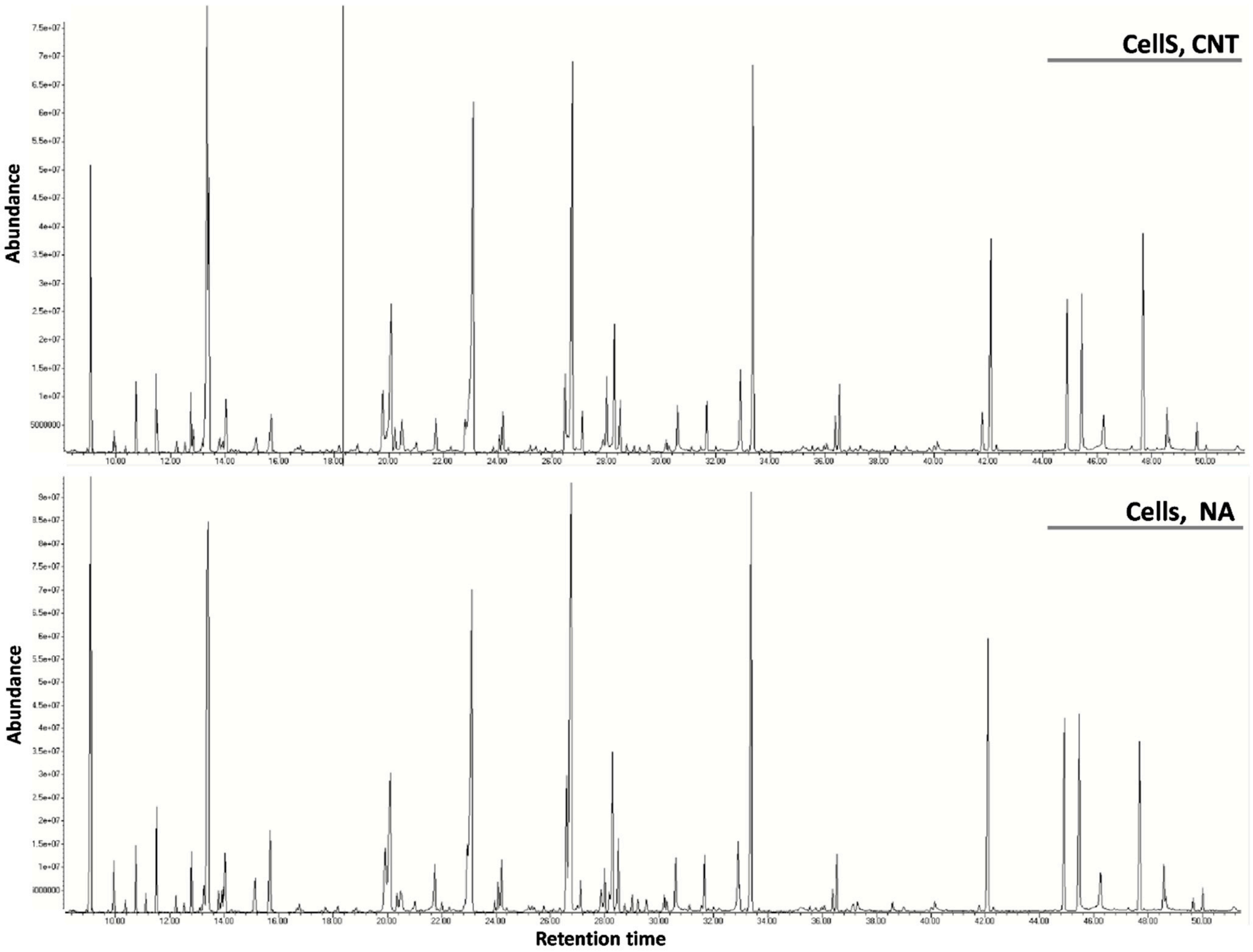

Figure S3. GC-MS chromatograms obtained from control (top) and Caco-2 cells treated with NA (bottom).

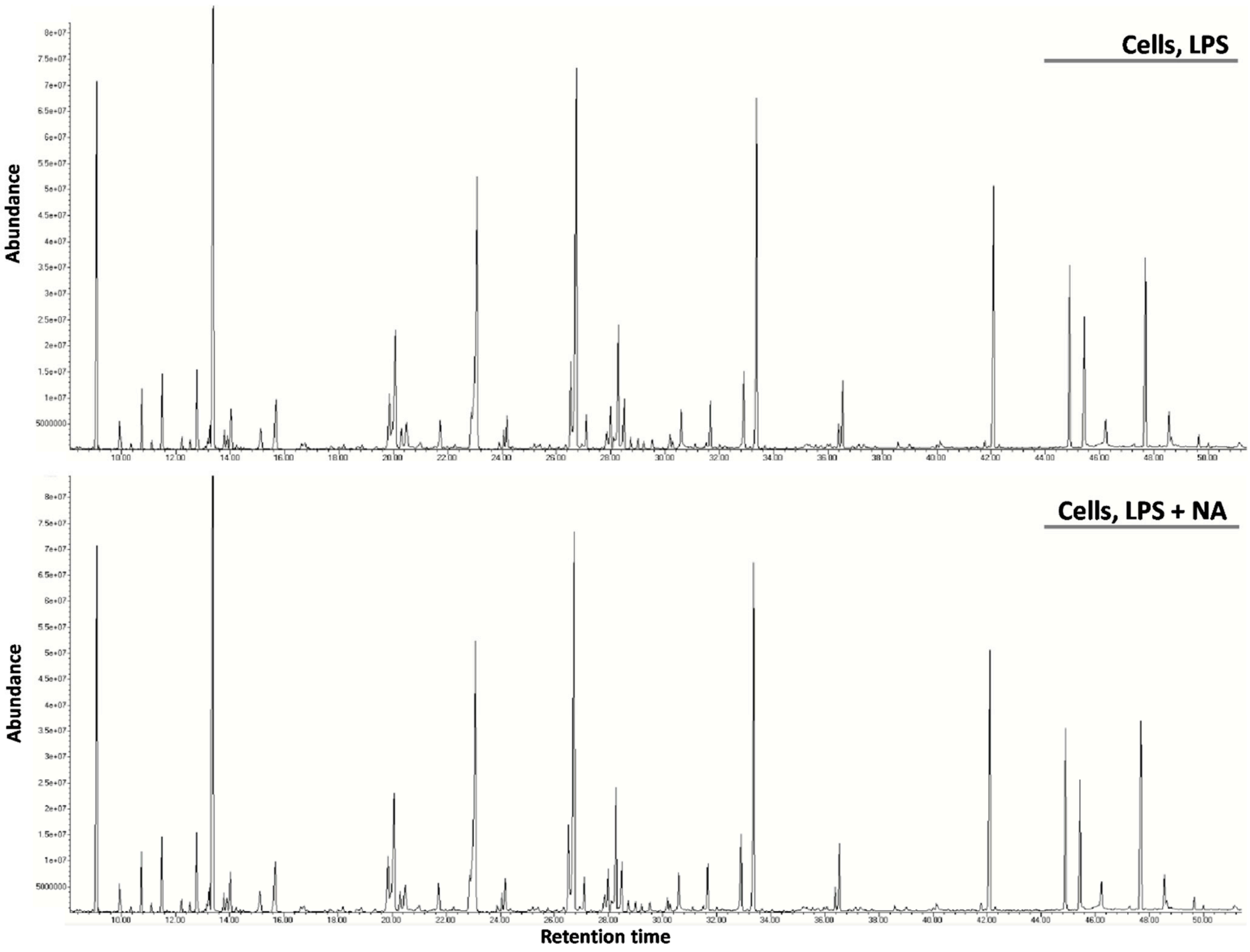

Figure S4. GC-MS chromatograms obtained from Caco-2 cells treated with LPS (top) and LPS+NA (bottom).

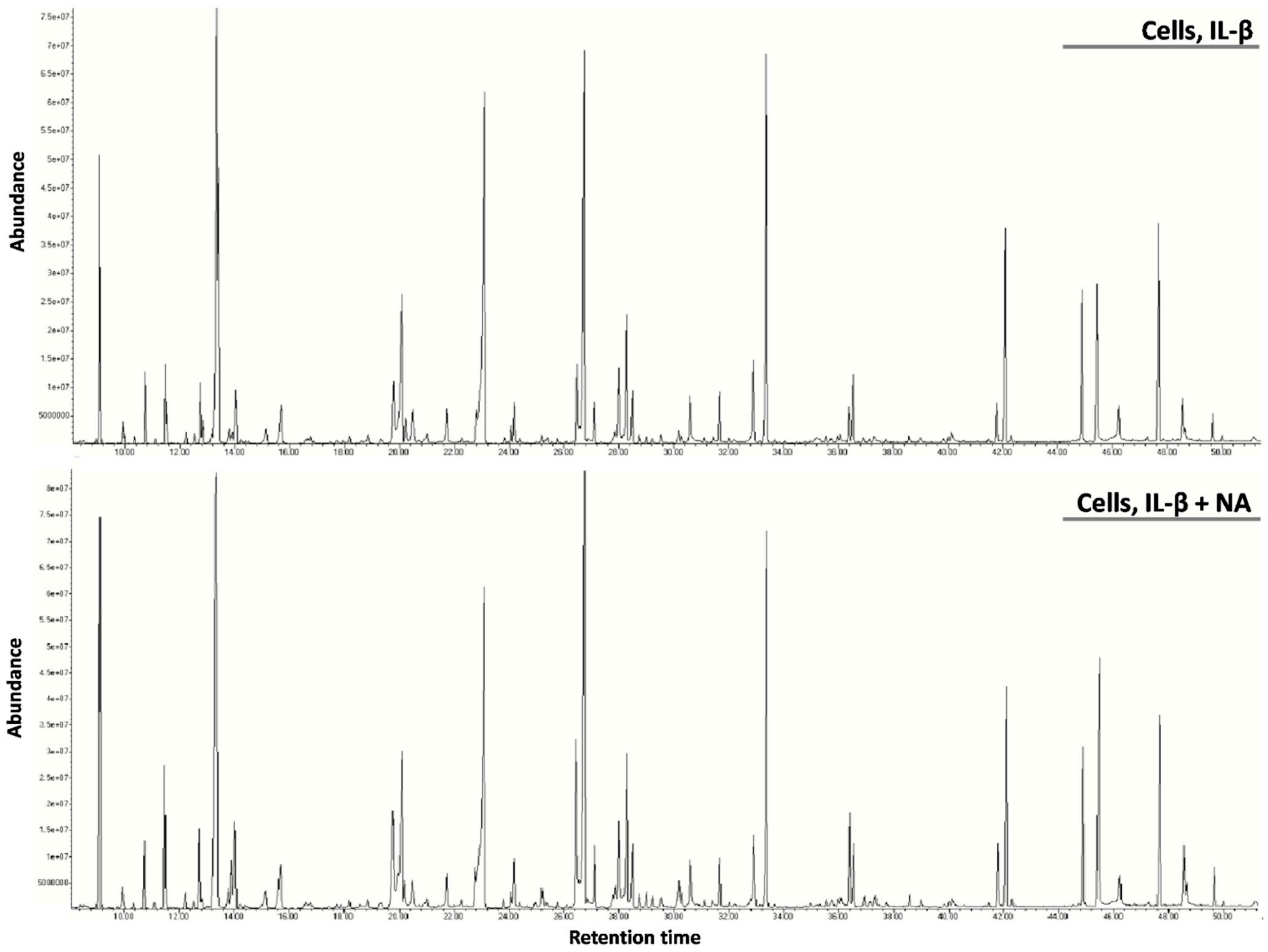

Figure S5. GC-MS chromatograms obtained from Caco-2 cells treated with IL-1β (top) and IL-1β+NA (bottom).

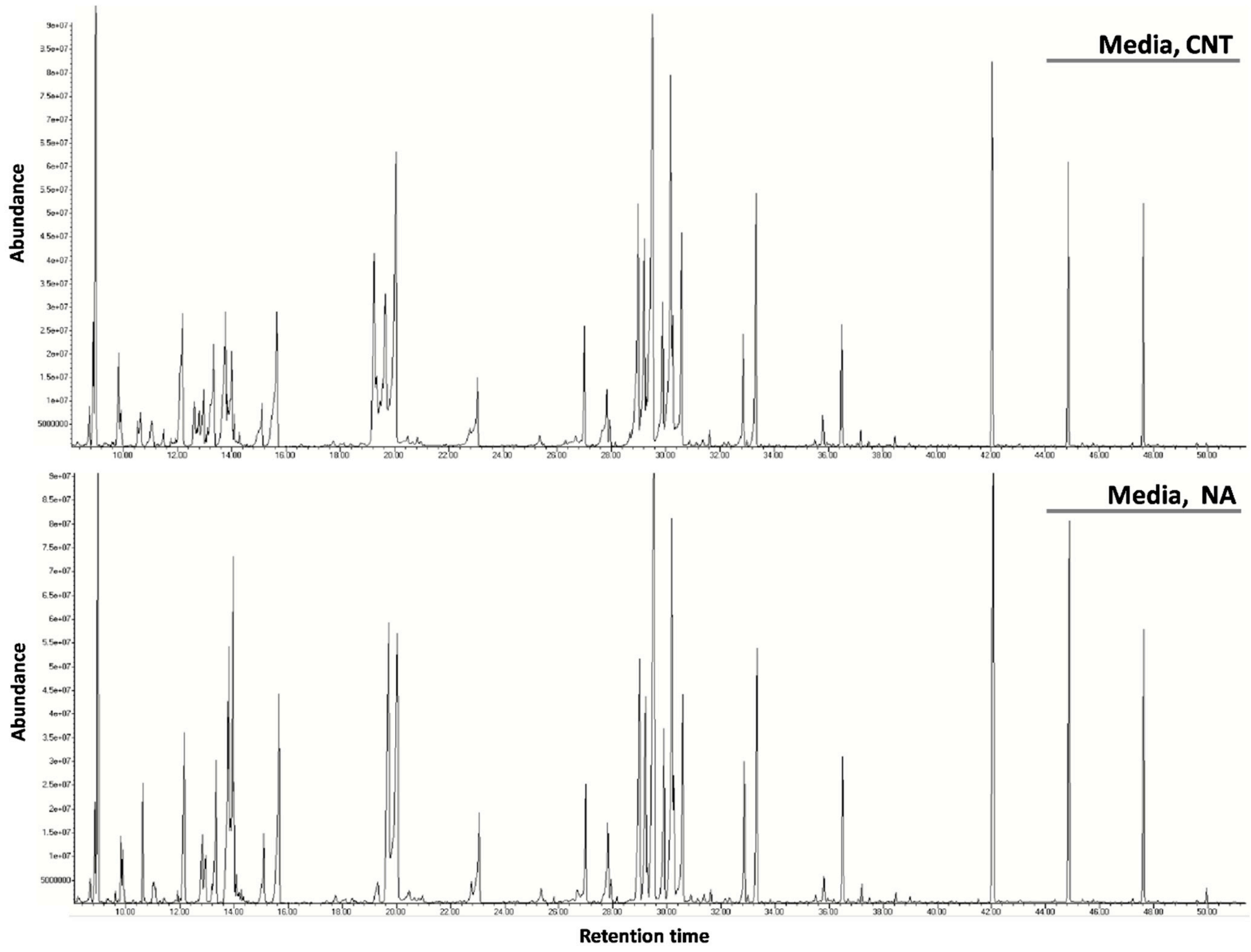

Figure S6. GC-MS chromatograms obtained from control Caco-2 cell culture media (top) and Caco-2 cell culture media after treatment with NA (bottom).

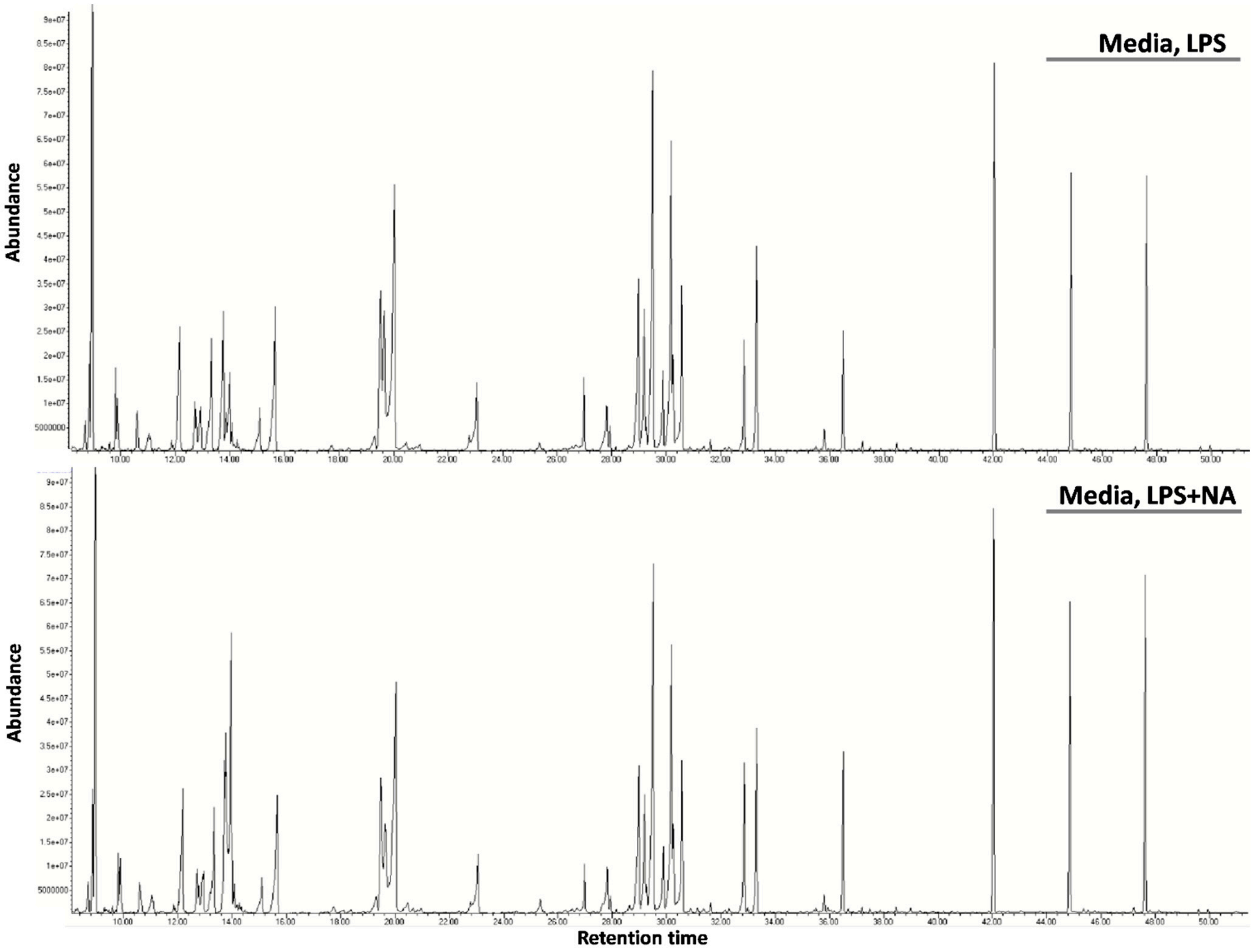

Figure S7. GC-MS chromatograms obtained from Caco-2 cell culture media after treatment with LPS (top) and LPS+NA (bottom).

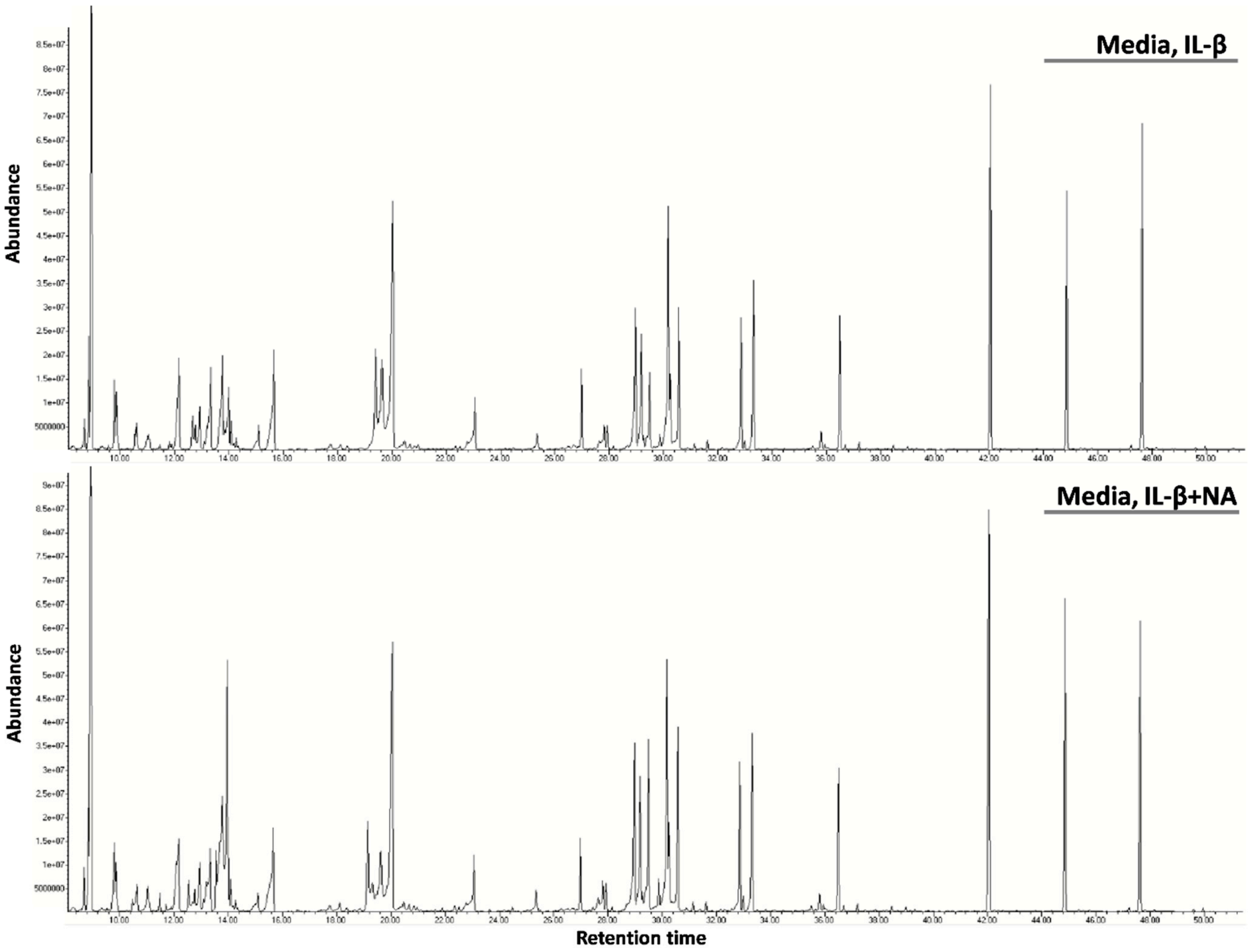

Figure S8. GC-MS chromatograms obtained from Caco-2 cell culture media after treatment with IL-1β (top) and IL-1β+NA (bottom).

Table S1. List of metabolites that were identified in Caco-2 cells and cell culture media by GC-MS analysis with each retention time (RT) and extracted ion (EI).

GC-MS metabolites			
**RT (min)**	**EI**	**Caco-2 cells**	**Caco-2 cell culture media**
11.11	147	3-Hydroxybutyric acid	3-Hydroxybutyric acid
48.56	169	Adenosine-5'-monophosphate	-
9.92	116	Alanine	Alanine
18.19	218	Aminomalonic_acid	Aminomalonic acid
16.88	160	Aspartic_acid	-
25.76	243	Beta-glycerolphosphate	-
49.65	129	Cholesterol	Cholesterol
27.99	273	Citric_acid	Citric_acid
21.01	220	Cysteine	Cysteine
12.53	174	Ethanolamine	-
29.00	307	Fructose	Fructose
15.13	245	Fumaric_acid	-
29.51	319	-	Glucose
20.48	174	Glutamic_acid	Glutamic_acid
21.84	147	Glutaric_acid	-
18.37	156	-	Glutamine
26.74	357	Glycerol-1-phosphate	-
14.03	174	Glycine	Glycine
22.27	188	Hypotaurine	-
32.19	318	Inositol	-
13.79	158	Isoleucine	Isoleucine
35.50	192	-	Kynurenine
9.09	147	Lactic_acid	Lactic_acid
11.09	86	Leucine	Leucine
25.32	156	Lysine	Lysine
18.86	147	Malic_acid	-
30.27	319	Mannitol	-
33.38	305	Myoinositol	Myoinositol
33.67	319	N-acetylglucosamine	-
18.95	179	Nicotinamide	-
27.11	299	O-phosphocholamine	-
27.86	142	Ornithine	Ornithine
31.68	291	Panthotenic_acid	Panthotenic acid
23.05	218	-	Phenylalanine
13.9	142	Proline	-
19.79	156	Pyroglutamic_acid	Pyroglutamic_acid
24.07	451	Pyrophosphate	-
8.94	174	Pyruvic_acid	Pyruvic_acid
15.13	204	Serine	Serine
30.25	205	-	Sorbitol
24.18	326	Taurine	-
19.32	205	-	Threitol
15.69	218	Threonine	Threonine

35.80	202	-	Tryptophan
15.28	255	Thymine	-
29.54	179	Tyrosine	Tyrosine
13.17	147	Urea	Urea
33.81	441	-	Uric_acid
45.46	169	Uridine-5'-monophosphate	-
12.22	144	Valine	Valine
